# The Acceptability and Experiences of Participating in a Preoperative Intentional Weight‐Loss Programme From Diagnosis to Surgery for Colorectal Cancer

**DOI:** 10.1111/hex.70896

**Published:** 2026-10-07

**Authors:** Amelia Talbot, Claire Foster, Susan A. Jebb, Pete Wheatstone, Simon Buczacki, Dimitrios A. Koutoukidis

**Affiliations:** ^1^ Nuffield Department of Primary Health Care Sciences University of Oxford, Radcliffe Primary Care Building, Radcliffe Observatory Quarter, Woodstock Road Oxford UK; ^2^ Centre for Psychosocial Research in Cancer: CentRIC, School of Health Sciences University of Southampton Southampton UK; ^3^ Patient and Public Involvement Member Leeds UK; ^4^ Nuffield Department of Surgical Sciences University of Oxford, Old Road Campus Research Building, Roosevelt Drive Oxford UK

**Keywords:** behaviour change, colorectal cancer, normalisation process theory, obesity, qualitative

## Abstract

**Introduction:**

Colorectal cancer surgery has a higher risk of complications for patients carrying excess weight. Preoperative weight loss might reduce this risk. However, it is unclear how patients perceive engagement with an intensive weight‐loss programme in the short window between diagnosis and surgery. This qualitative study, embedded within the CARE randomised controlled trial, aimed to understand the acceptability and experiences of following an intensive weight‐loss programme with dietetic support as prehabilitation before colorectal cancer surgery.

**Methods:**

Thirty‐one participants (65% male, mean (SD) BMI 36.0 (5.5) kg/m^2^, 65 (7) years) took part in a semi‐structured interview based on the Theoretical Framework of Acceptability after following the programme for an average of 24 (12) days and 12 (7) days before surgery. Data were thematically analysed using the four constructs of Normalisation Process Theory (NPT).

**Results:**

Participants understood, via interactions with healthcare professionals, that the programme could potentially make their operation technically easier and expedite hospital discharge (coherence). Continued adherence was supported by the dietitians (cognitive participation), and improvements in health markers and rapid weight loss helped to reframe some participants' views that their bodies were failing due to cancer (reflexive monitoring). However, some families' distrust of the programme's safety, social eating occasions, and side effects prevented full acceptability and total adherence (collective action). This highlighted how collective action sometimes overrode the consistently strong coherence, cognitive participation, and reflexive monitoring components of NPT.

**Conclusion:**

The weight‐loss programme was mostly acceptable to people who had already joined the intervention. Addressing challenges with collective action may support more patients to adhere.

**Clinical Trial Registration:**

ISRCTN39207707, Registration date 13/03/2023.

## 1 Introduction

Colorectal cancer is the third most common cancer worldwide [1]. Two‐thirds of those diagnosed carry excess weight, of which half have obesity [2]. Most patients will undergo colorectal resection surgery as their initial treatment [3]. However, excess weight independently increases the postoperative morbidity risk [4, 5, 6]. Intentional weight loss before surgery might reduce this risk [7]. In England, surgery is expected to occur within 31 days following the decision to treat [3]. This short timeframe, therefore, requires an intensive intervention to achieve meaningful weight loss.

One such potential intensive intervention is a low‐energy total diet replacement (TDR) programme with behavioural support. During TDRs, individuals substitute all foods with high‐protein nutritionally complete formula products providing ~800 kcal/day while they are supported by trained counsellors [8]. TDRs are a cost‐effective treatment for obesity [9] and have resulted in significant weight loss in diverse populations with obesity‐related diseases [10, 11, 12]. People receiving a TDR programme as treatment for type 2 diabetes within the healthcare system have also reported finding this intervention acceptable [13, 14, 15, 16].

The CARE randomised controlled trial (ISRCTN39207707, registration date 13/03/2023) explored the feasibility of intentional weight loss using a TDR with dietitian support as prehabilitation before colorectal cancer surgery [17]. The full trial results have been published elsewhere [18]. To summarise, CARE found that recruitment and retention to the trial alongside engagement and adherence to the intervention were feasible. Intervention participants attended 85% of their expected dietetic calls. Most of those providing feedback (79%) reported that the intervention met or exceeded their expectations and participants were almost twice as likely to report that it took no or a little effort to follow the diet compared to a lot or a huge effort. Intervention participants lost an average (SD) of 6.1 (3.0) kg over the month before surgery, which was 4.3 kg (95% CI, 2.7–5.8 kg) more than usual care participants. There was no evidence of between‐group differences in complications (14 [39%] vs. 14 [40%]), but those who lost 3.2% or more of their body weight (above the median loss) had a 50% relative reduction in complications. While these findings support the safety and feasibility of TDR for a definitive trial, patients' experiences of following this intensive intervention during a psychosocially distressing time remain unexplored [19]. Understanding these perspectives in depth will help to refine the intervention to potentially increase acceptability and support adherence in the definitive trial.

The wider literature has shown that self‐efficacy might impact a patient's ability to engage in prehabilitation. For example, a systematic review highlighted that the shock and anxiety experienced after a cancer diagnosis can make the idea of prehabilitation feel overwhelming [19]. Symptoms of cancer can also overlap with potential side effects of prehabilitation weight‐loss and exercise interventions [20, 21]. Clinicians have had concerns that these symptoms, combined with the short period from diagnosis to surgery, could limit patients' self‐efficacy to engage [22]. Conversely, some patients have reported that their cancer diagnosis increased self‐efficacy by creating a window of opportunity to take control over their health and surgical outcomes and acted as a stimulus for further healthy dietary changes after surgery [23, 24]. Health literacy may also play a key role, as patients need to understand information on how weight loss might improve surgical outcomes to make an informed decision about following a TDR [19].

This qualitative study aimed to understand the acceptability and experiences of following an intensive weight‐loss programme with dietetic support as prehabilitation before colorectal cancer surgery as part of the CARE trial.

## 2 Materials and Methods

### 2.1 Context and Design

CARE recruited 71 patients across eight academic and community hospitals in England between March 2023 and August 2024. Participants had a BMI ≥ 28 kg/m^2^, were awaiting curative colorectal cancer resection, and had at least 20 days until their surgery [17]. Patients were excluded if they reported a weight loss of ≥ 10% in the 6 months prior to screening. Following 1:1 randomisation to usual care or TDR, intervention participants replaced all food with four meal replacement products per day until their surgery. The products included soups, shakes, and porridge (~ 800 kcal/day, ~76 g protein/day) [25]. Intervention participants also had weekly dietetic calls. Ethical approval was granted by the South Central – Oxford B Research Ethics Committee (Ref22/SC/0465).

### 2.2 Recruitment and Sampling

Thirty‐eight participants were allocated to the intervention and invited to an interview for maximum variation regardless of intervention adherence [26].

### 2.3 Data Collection

AT (female medical sociologist) contacted participants over email/telephone to arrange the interview. We aimed to interview ~2 weeks after patients started the TDR and at least a week before surgery on advice of PW, who has lived experience of colorectal surgery, to minimise burden. The interviews were one‐to‐one, semi‐structured, and over telephone using the MS Teams function [27]. Interviews were led by AT (*n* = 26) or DAK (*n* = 5; male chief investigator, dietitian uninvolved with intervention delivery). They regularly discussed areas to explore in subsequent interviews. Neither knew the participants before the interview, and participants were aware they were part of the trial team.

Questions were informed by the Theoretical Framework of Acceptability [28], similar questions asked of participants following TDRs in other settings [14, 15, 16, 29], expertise of co‐authors in cancer (S.B.) and/or weight management programmes (D.A.K., S.A.J., C.F.), and our public contributor (P.W.) (Supporting Information S1: File 1). Questions were added as needed to explore unanticipated areas raised by participants (e.g., interpersonal challenges to adherence). Notes were taken during the interviews to inform follow‐up questions and afterwards to promote reflexivity around any assumptions we had towards the data collected [30]. The guide was not shared with participants.

Interviews were audio‐recorded (mean (SD): 31 (15) minutes, range 10–68) and transcribed verbatim. Transcripts were not checked by participants.

### 2.4 Data Analysis

AT analysed the data using inductive thematic analysis [31]. Her ontology was critical realist [32]. AT listened to audio recordings, manually noted codes for each line of data, and then merged codes into broader descriptive themes. DAK re‐coded a random 10% of the data set. Results were discussed with AT to identify overlooked elements and reflect on alternative and additional interpretations.

AT then conducted a theory‐driven analysis using ‘Normalisation Process Theory' (NPT), which has previously been used in prehabilitation studies of patients undergoing cardiac surgery [33]. NPT is a sociological framework for understanding the individual and collective ‘work' involved to enable complex interventions to become routine (normalised) in healthcare [34]. Four interrelated constructs act as facilitators or barriers to this process throughout the intervention (Table 1). NPT was chosen because we considered the intervention as a new practice that could require significant behavioural change from participants. NPT provided a comprehensive framework to organise and critically reflect on this.

**Table 1:** The constructs and components of normalisation process theory.

Construct	Components
**Coherence:** The ***sense‐making work*** to understand the purpose and potential benefits of the intervention.	**Individual Specification:** Participants' understanding of what they need to do as part of the intervention. **Communal Specification:** Understanding within participants' social networks of the value and importance of the intervention. **Internalisation:** Participants' understanding of the value and importance of the intervention. **Differentiation:** Participants' ability to distinguish the intervention from their previous dietary habits and other diets.
**Cognitive Participation:** The ***relational work*** to build and sustain motivation and adherence to the intervention.	**Initiation:** The actions taken by health care providers to promote participants initial engagement with the intervention. **Enrolment**: The process by which participants organise and reorganise themselves with their healthcare providers to promote adherence to the intervention. **Activation:** The ongoing actions by participants and their healthcare providers to maintain motivation and adherence to the intervention **Legitimation:** The ongoing process of establishing credibility and acceptance of the intervention as part of prehabilitation among participants with their healthcare providers and their families.
**Collective Action:** The ***operational work*** to enact the intervention into everyday practice.	**Interactional Workability:** Participants' interactions with the programme that may enable or hinder continued adherence. **Relational Integration:** The knowledge between patients and their families that the programme is safe and effective. **Skill‐Set Workability:** The allocation of tasks to build participants' adherence to the programme. **Contextual Integration:** Participants' understanding of how the programme fits within their social and environmental contexts.
**Reflexive Monitoring:** The ***appraisal work*** to evaluate whether the intervention had been beneficial and effective.	**Individual Appraisal:** Participants' evaluation of whether the intervention had been beneficial and effective. **Communal Appraisal**: The evaluation by people within participants' social networks of whether the intervention had been beneficial and effective for the participant. **Systematisation:** The process of collecting and analysing data to assess the effects of the intervention. **Reconfiguration:** Participants' recommendations on how the intervention could be modified to improve its acceptability and increase adherence in the future.

AT used the ‘One Sheet of Paper' method [35] to manually map all data, codes, and their relationships against the NPT toolkit to create four analytical themes for each construct [36]. This included data from one participant who did not commence the TDR but shared a written reflection on intervention acceptability and provided weight measurement data at 30 days follow‐up. AT did not identify any data inconsistent with NPT, suggesting the framework was an appropriate fit for our data set. She also determined that saturation was achieved because the constructs and their relationships were fully developed and no additional experiences were discussed by the final few participants [37].

### 2.5 Trustworthiness and Reflexivity

We used several techniques to increase the trustworthiness and credibility of our analysis. A.T. had multiple coding clinics with D.A.K. and C.F. (female, Professor of Psychosocial Oncology) to discuss and refine ongoing codes and interpretations. Our diverse disciplinary backgrounds and varying levels of familiarity and expertise in the topic area allowed us to explore different understandings of the data, challenge interpretations and underlying assumptions, and highlight nuances that might have otherwise been overlooked by a single researcher. Final constructs were reviewed by all study authors, including P.W., as a form of member checking to ensure our findings reflected the patient's lived experience.

AT maintained reflexivity of her positionality, engaging critically with how her assumptions, personal beliefs, and experiences may have impacted the data generation process and why [30]. She approached the study as an outsider, having no professional or personal experience with oncology or weight management programmes but having expertise in mental health. Her initial assumption was that few patients would adhere to the programme during a time when food can become an emotional comfort. As such, she was naturally inclined towards asking participants and looking at the data on how the programme impacted people psychosocially. This was also why the coding clinics and using NPT as a theoretical lens was helpful, encouraging AT to look at other parts of participants' experiences.

## 3 Results

Thirty‐one out of 38 participants randomised to the intervention group were interviewed: 27 who completed the intervention, one who completed the intervention but elected not to have surgery, and three who withdrew after commencing the intervention. The average (SD) time from commencement to interview was 24 (12) days (range: 4–50). The average time from interview to surgery was 12 (7) days (range: 4–33). Figure 1 shows the flow of participants through the study, including reasons for non‐interview. Table 2 shows that the participants' demographics were comparable to those of the overall trial intervention group.

**Figure 1:**
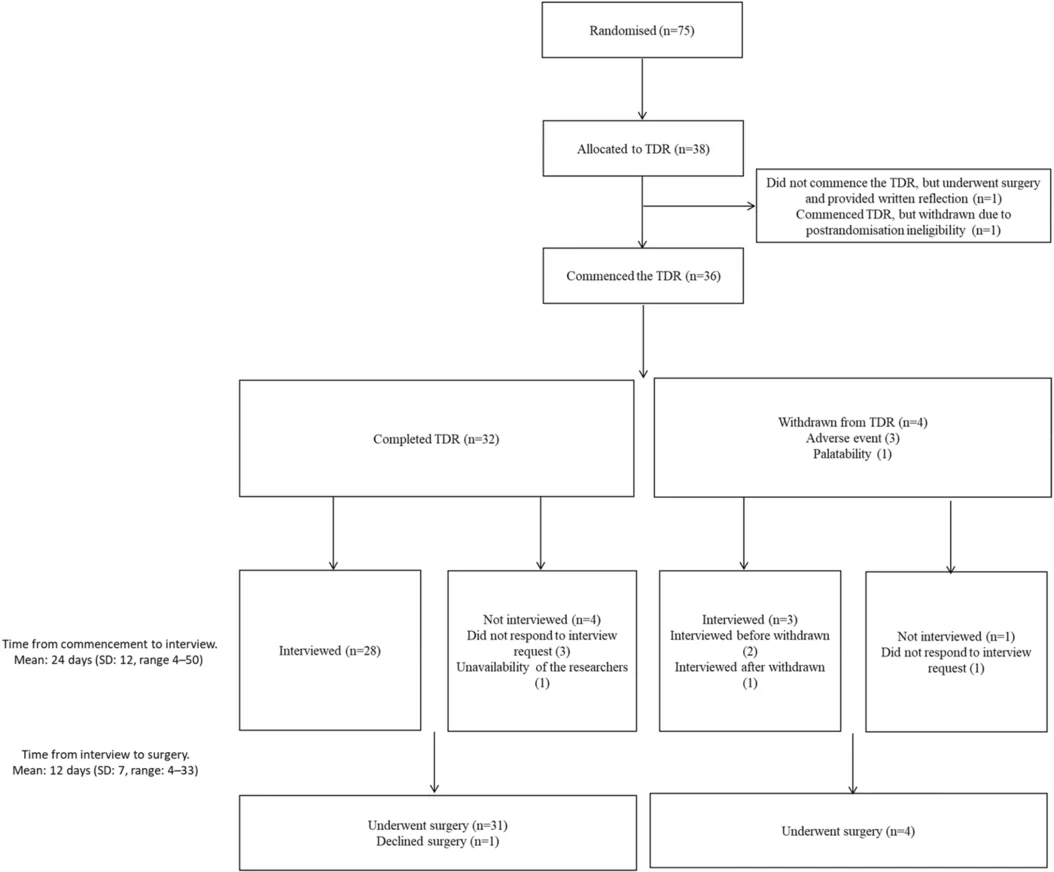
Flow of participants through the study.

**Table 2:** Participants' demographics (*n* = 31) compared with trial intervention Sample.

Participant Demographics	Interview Sample *N* (%) (*n* = 31)^a^	Trial Intervention Sample *N* (%) (*n* = 36)^b^
Ethnicity		
White	29 (94)	34 (94)
Black/Other	2 (6)	2 (6)
Sex		
Male	20 (65)	25 (69)
Female	11 (35)	11 (31)
Index of multiple deprivation (IMD)^c^		
1–2 (least advantaged)	4 (13)	3 (8)
3–4	5 (16)	5 (14)
5–6	5 (16)	7 (19)
7–8	8 (26)	9 (25)
9–10 (most advantaged)	9 (29)	12 (33)
Recruitment area		
South East	10 (32)	11 (31)
East	8 (26)	11 (31)
Yorkshire and the Humber	4 (13)	4 (11)
South West	3 (10)	4 (11)
West Midlands	3 (10)	3 (8)
East Midlands	3 (10)	3 (8)
Age, years	Mean, standard deviation	
	65 ± 7	65 ± 7
Period from randomisation to admission, days	36 ± 12	37 ± 17
Baseline weight, kg	105.0 ± 16.7	104.4 ± 17.0
Baseline BMI, kg/m^b^	36.0 ± 5.5	35.5 ± 5.5
Weight on admission, kg	99.2 ± 16.9	98.3 ± 16.1
Weight change, kg	−6.7 ± 2.8	−6.1 ± 3.0

^a^ The interview sample includes one participant excluded from the trial intervention analysis due to declining surgery upon completion of the weight loss programme.

^b^ Two of the 38 randomised participants were excluded from the trial analysis due to post‐randomisation eligibility or due to declining surgery.

^c^ IMD is the official measure of relative deprivation in England. The indices rank 33,755 small areas from most to least deprived and then divide them into ten equal deciles (available: https://deprivation.communities.gov.uk/).

The four themes are organised below using the NPT construct headings. Theme four is divided into two sub‐themes to show participants' appraisal of the weight loss programme and their evaluation of pre‐defined modifications to the intervention for future trials. Quotes are attributed to participants by sex, age, BMI at baseline, and weight loss achieved between randomisation and admission for surgery (kg). Additional quotes are in Supporting Information S2: File 2.

### 3.1 Construct One (Coherence): Healthcare Professionals’ Sense‐making Work Acted as a Catalyst for Participants’ Understanding of the Potential Importance of the Intervention

Participants demonstrated an understanding that the TDR involved using four meal replacement products per day until their surgery. This understanding was underpinned by the belief that significant weight loss could directly improve their surgical outcomes; indeed, several participants perceived that every pound lost would have a cumulative benefit to their operation:

It's definitely worthwhile if you're going in for an op and you can lose this amount of weight because every extra bit of weight you lose is a positive for your operation.

(Female, 56 years, BMI 33.4, −6.7 kg)

Participants made sense of the intervention by differentiating the TDR from previous weight‐loss attempts. Their view was that the intervention offered a structured and simple pathway to rapid and significant weight loss, which contrasted with their prior experiences where progress had been comparatively slow:

It's such a restricted diet. I've never tried going down the route of just having shakes and soups. I think that's what's making a difference, and I have lost quite a bit in the first week.

(Male, 69 years, BMI 37.6, −6.6 kg)

However, the primary reason participants understood the intervention's purpose and potential benefits was the sense‐making work of healthcare professionals prior to and at the start of the intervention. For many, this professional input served as the catalyst that transformed a general awareness of their overweight into an understanding of why the intervention might be necessary for their surgery:

The motivation is because the doctor said, ‘You need to lose some weight. It will aid you in the operation and also in the recovery'. I knew I was overweight. But that sunk in when he said that to me.

(Male, 59 years, BMI 38.2, −8.1 kg)

Participants learned through conversations with surgeons specifically that losing weight could make their surgery technically easier by reducing visceral fat and facilitate an earlier discharge due to improved wound healing. One participant described hoping that being home sooner would reduce his risk of infection and improve his mental health:

He's [surgeon] not had to cut through as much. It means that there's less to repair. It means I come home as soon as possible. I'm less likely to get an infection when I'm at home… Being in my home mentally, I'm also going to feel better.

(Male, 62 years, BMI 38.6, −2.3 kg)

However, one participant who provided a written reflection had a different view. After randomisation and prior to commencing the intervention, this participant, alongside his wife and colleagues, perceived that he had lost too much weight to be eligible for the trial. Despite the trial team re‐confirming his eligibility through his records, this process of collective sense‐making led him to withdraw from the intervention:

I do not think a programme aimed at weight loss is appropriate… One of the identifying factors of my cancer is rapid weight loss. In July, I weighed 87.5 kg, and I weighed 78 kg [in December], a loss of 10.8% and still losing it.

(Male, 64 years, BMI 28.3, −2.4 kg)

### 3.2 Construct Two (Cognitive Participation): The Relational Work Between Healthcare Professionals and Participants Was an Important Mechanism for Encouraging Intervention Adherence

Once participants initiated the intervention, healthcare professionals' sense‐making work transitioned into relationally working with the participants to sustain their motivation and encourage adherence. For example, the research nurses had calls with participants as they moved through the intervention. A couple of participants perceived these interactions as offering reassurance that they were not alone during the TDR, but were part of a supported, collective effort:

Everybody at the hospital has been very supportive. I've had the cancer nurse on this morning supporting me. It's just that knowledge there's somebody there if you need to speak to somebody or come back to you and support you in your effort.

(Male, 69 years, BMI 37.6, −6.6 kg)

The dietetic calls were perceived as central to this relational work. Almost all participants described that these calls made them feel accountable and helped solve practical challenges; this included managing constipation and, most frequently, incorporating flavourings and crudités, as many missed the texture of solid food, found some products bland, and some experienced mild hunger:

I spoke to the dietitian, and she said I could have celery and raw carrots. I've started to do that a little bit if I get hungry. It also helped with the fibre because I was having problems with constipation.

(Female, 56 years, BMI 33.4, −6.7 kg)

The relational work of the dietitians was also evident in their support of participants who temporarily lapsed from the TDR. Participants reported having a strong rapport with their dietitians, which created a non‐judgmental environment where they could openly discuss these lapses and receive advice and encouragement to resume the intervention:

I felt as if I could tell her [dietitian] when I had cheated, that I had a ham sandwich… She was really understanding, and she said, ‘We're all bound to have a few blips now and then.

(Female, 59 years, BMI 35.8, −5.5 kg)

Similarly, two participants who had challenges fully adhering due to side effects but wanted to continue for the surgical benefits found it helpful to work with their dietitian on a tailored plan. This included the following participant who experienced dizziness from a viral infection and believed he required at least one meal alongside the TDR to be fit for surgery:

[The dietitian advised me to] basically carry on doing what I'm doing… What I'm trying to do is to do two or three of the shakes a day and then have something. For example, tomorrow, I might have salmon with some vegetables.

(Male, 62 years, BMI 38.6, −2.3 kg)

### 3.3 Construct Three (Collective Action): The Operational Work of Enacting the Intervention Was Affected by Family Attitudes, Social Eating Occasions, and Side Effects

Participants were motivated to follow this programme due to the potential benefits to their surgery, ongoing problem‐solving support from dietitians, and improvements in their health (construct four). However, we understood that the operational work to enact the programme sometimes overrode these motivators. These challenges were categorised into three areas, the first was the difficulty of adhering during social eating occasions, including birthdays and Christmas. For example, the participant below said she was committed to the intervention due to its positive impact on her diabetes and upcoming surgery, yet accepted eating other foods during special social events. Many others reported following the intervention due to the potential surgical benefits, but found that eating other foods during social occasions was important for maintaining normalcy and preserving social bonds:

It was my choice to have the ham sandwich and a small slice of cake just to satisfy myself. I would take on the consequences because it was the grandchildren's birthday. I just thought I'd treat myself. It wasn't going to happen very often, and I didn't beat myself up about it.

(Female, 59 years, BMI 35.8, −5.5 kg)

This was also observed when we spoke with participants about preparing meals and eating with other household members. While some participants were tempted by the smells but successfully abstained, others reported either eating small portions of these foods or taking measures to avoid doing so, such as leaving the room while their family ate or leaving the house to go for a walk:

I went to the to the local pizza cafe and bought a pizza for them [his child and their friends], brought it back [home]. And I said there you go boys, you have the pizza. I'm going to go for a walk because the smell is driving me crazy.

(Male, 61 years, BMI 38.2, −13.3 kg)

The second aspect of this overriding was when families questioned the safety of the intervention. On one hand, most families were reported to have supported adherence by sharing encouraging words, engaging in their own weight loss attempts, or removing temptations from the home. We interpreted this as reinforcing the initial sense‐making and ongoing relational work that framed the intervention as potentially benefiting surgical outcomes:

She's [wife] also done a very similar regime to the one I've been on over the last 6 weeks. It's definitely helpful having somebody doing it with you because they're not going to put anything tempting in your way.

(Male, 61 years, BMI 32.4, −7.8 kg)

On the other hand, some other participants said their families commented that they had been on the programme for too long, appeared unwell due to their rapid and evident weight loss, and suggested they could withdraw at any time if they experienced side effects:

It doesn't help when they [family] start saying, ‘You don't want to lose any more weight. People are starting to think you look ill.

(Male, 71 years, BMI 29.2, −10.4 kg)

Participants said they were not discouraged by these comments, as they had engaged in the sense‐making work to understand the potential of the programme for their surgical outcomes and were reassured that they could relationally work through any concerns with the dietitians. However, a few participants made modifications or stopped following the programme as a result, highlighting again how collective action sometimes worked against the other constructs and prevented adherence:

My husband said, ‘How can you suffer like that'? I said,' Because I need to lose weight.' And he went, ‘You're starving to death. You're going to get malnutrition.' He said to make sure I've got enough nutrition, protein, minerals, and vitamins and add something instead of your shake or your porridge. That's what I've been trying to do.

(Female, 62 years, BMI 33.9, −12.2 kg)

The final way this overriding prevented adherence for a few participants was side effects. While these participants had sense‐made the potential importance of weight loss for surgery, the symptoms that came with enacting the programme like dizziness and hunger meant they stopped following it. Instead, these participants opted for alternative, self‐directed weight loss behaviours:

I totally agree with all the reasons for it [intervention]. But I didn't realise overall how it would affect my blood sugars and my hunger. I was going to bed hungry all the time, and the feeling you get when your blood sugar drops… So, I'm cutting down my meal portions and trying healthier options like no fat.

(Male, 66 years, BMI 30.2, −2.2 kg)

However, participants expressed not feeling as hungry as they had expected. They reported that hunger decreased after a few days and those who still experienced it found it tolerable. Others reported tiredness and dizziness, but these also mostly resolved after a few days. Participants generally attributed these effects to their bodies adjusting to the significant reduction in dietary energy intake:

I kind of assumed that because of the limited intake, feeling hungry would be a problem. But that was not as bad as I thought it would be. You're not actually that hungry.

(Male, 61 years, BMI 32.4, −7.8 kg)

### 3.4 Construct Four (Reflexive Monitoring): Participants’ Appraisal Work Identified That the Rapid Weight Loss They Experienced Could Positively Impact Their Surgery, While Also Recommending Increased Product Variety to Prevent Monotony and Promote Cultural Inclusivity

#### 3.4.1 Sub‐Theme One: Appraisal of the Intervention

We asked participants to give an overall appraisal of how the TDR had impacted their weight and whether they felt these changes could influence their surgical outcomes. We understood that most participants provided a positive appraisal, which they tied to the rapid and tangible results of weight loss they experienced in a relatively short timeframe:

I'm very confident [that the TDR will benefit my surgery] because I've lost a reasonable amount of weight in a relatively short space of time.

(Male, 76 years, BMI 49.2, −7.5 kg)

Coinciding reported improvements in energy, mobility, and breathing also gave participants confidence that the intervention had optimised their functional capacity for surgery. For those asked to self‐monitor, improvements in blood pressure and blood glucose added to their perceived positive appraisal of the TDR. These changes created a positive feedback loop for participants throughout the intervention; the tangible results reinforced the potential benefits of the TDR that had been established during their initial sense‐making with healthcare professionals:

My blood pressure has come down. I was on two tablets for probably ten years. I've been taking my blood pressure every day, and about two weeks ago, it had noticeably come down. She [dietitian] suggested if it continued coming down to stop one of the tablets, which I have done. It would be nice if that continued.

(Female, 72 years, BMI 34.0, −5.1 kg)

These positive impacts on physical health extended to some participants' mental health. One participant described a shift from feeling powerless and lacking control over her body to viewing her body more positively due to being engaged in an intervention that she appraised as benefiting her physical health and potentially her surgery:

It [TDR] distracts me because sometimes, with the illness, you feel your body's failing you. I used to have joint aches because I added weight during menopause. I don't have that anymore… It's a distraction from feeling my body failing to feeling like my body's looking better. I look better in clothes. I'm not so tight.

(Female, 54 years, BMI 34.8, −7.9 kg)

Participants were also asked to appraise how the intervention could be changed to improve its acceptability for future patients with colorectal cancer undergoing surgery. They reported finding the taste of the products mostly acceptable, though a few participants had criticisms about their grainy texture, the small variety of breakfast options, and the sweetness of milkshakes. One participant said this led him to self‐manage his weight loss by adhering to 800 kcal using alternative foods rather than the TDR:

I'm used to a solid diet. I'm not six months old. I can't stand the porridge. I can't have anything hot for breakfast. The sweet things I find too artificial… I've not stuck to the product. I've stuck to a diet, but not the product.

(Male, 72 years, BMI 28.7, −9.1 kg)

However, there was a consensus that the TDR became monotonous over time and that more variety would be beneficial. Some also suggested adding a meal replacement bar in place of the soups and shakes and being explicit about crudités being allowed alongside the TDR to help participants who missed the texture of solid food. One participant from an ethnic minority background raised the point that the products were catered primarily to the “*British culture of food*” and could benefit from being tailored more to patients from different cultures:

It's [TDR] based on the British culture of food. I've never had porridge in my life, I've never had shakes… I'm from [North Africa]. So, it's totally different things we eat, and I had to get used to the things [dietary products]. I think maybe you can touch base with other cultures.

(Male, 58 years, BMI 29.1, −4.3 kg)

### 3.5 Sub‐Theme Two: Appraisal of Potential Modifications to the Intervention for Future Trials

Participants were asked to appraise pre‐defined modifications to the TDR for future trials, such as extending the intervention period to 6 weeks. In response, participants had concerns about their cancer progressing:

At the moment, it [cancer] hasn't spread, but as the consultant said, the longer it's there, the more possibility there is of it spreading. We don't even know what stage it is until it's cut out. So no, I wouldn't want to delay it [surgery] at all.

(Male, 66 years, BMI 30.2, −2.2 kg)

For reinitiating the TDR after recovering from surgery, participants were open to the idea because of concerns about weight regain once the intervention and dietitian support ended. However, participants also explained that without the potential surgical benefits driving their motivation, and given the social limitations imposed by the TDR, they might not adhere as closely:

I can't say whether I would stick to it 100% [following the TDR after recovery from surgery]. I have had to turn down sort of social dinners and things like that with friends because obviously I'm having a shake, and they're going out for a beer and curry.

(Male, 61 years, BMI 38.2, −13.3 kg)

In terms of the integration of exercise with the TDR, participants were divided. Some were unsure of its feasibility due to the impact of comorbidities, while others viewed the combination as an acceptable way to maximise weight loss and further optimise surgical recovery. A common concern was that the deficit from the TDR, combined with exercise, might increase fatigue:

At the moment, I would find that difficult… I've had hip surgery not long ago. I haven't fully recovered from that. We're also in the process of moving house. Extra things on me at the moment just doesn't work.

(Male, 72 years, BMI 28.7, −9.1 kg)

## 4 Discussion

### 4.1 Summary

This qualitative study showed that an intensive weight‐loss programme as prehabilitation before colorectal cancer surgery was interpreted to be mostly acceptable to participants who had already chosen to join the trial. Participants understood, via interactions with healthcare professionals, that the programme could potentially make their operation technically easier and expedite hospital discharge (coherence). Continued adherence was supported by the dietitians (cognitive participation), and improvements in health markers and rapid weight loss helped to reframe some participants' views that their bodies were failing due to cancer (reflexive monitoring). However, some families' distrust of the programme's safety, social eating occasions, and side effects prevented full acceptability and total adherence (collective action). We understood these experiences as showing how collective action sometimes overrode the consistently strong coherence, cognitive participation, and reflexive monitoring components of NPT (Figure 2).

**Figure 2:**
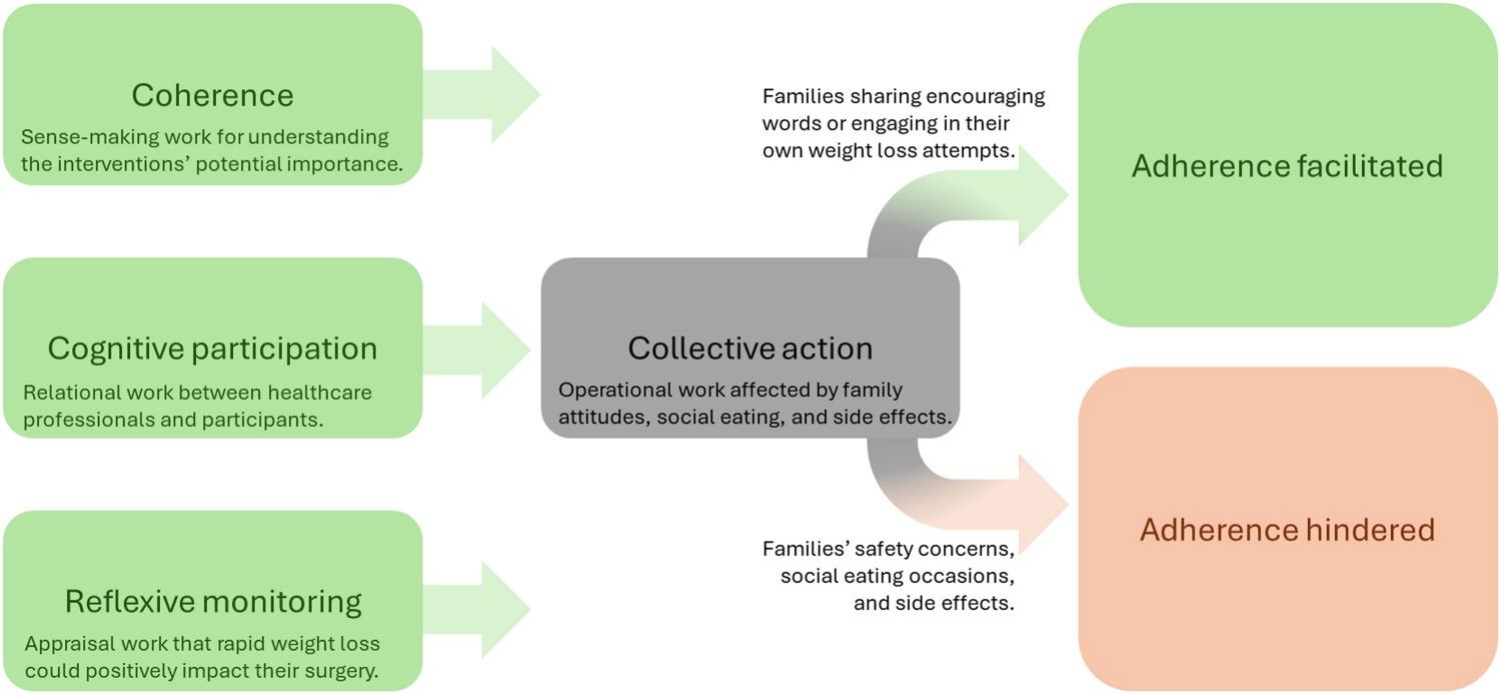
Illustration of NPT components supporting and hindering acceptability of the weight loss programme.

### 4.2 Context With Other Literature and Implications

Results of a qualitative systematic review showed that the anxiety experienced following a cancer diagnosis can act as a barrier to prehabilitation [19]. While participants frequently expressed feelings of anxiety related to their diagnosis during a separate study on their experiences of being recruited to this trial [38], they commonly described participating because of the potential benefits to their surgery. Interestingly, some participants also reported that the intervention reframed their perception that their bodies were failing due to cancer. This suggests that most trial participants might have had high self‐efficacy towards their ability to participate in the intervention.

Our data suggest that most trial participants might have had high self‐efficacy towards their ability to participate in the intervention. Like prehabilitation studies involving dietary change for other cancers [38, 39, 40], self‐efficacy was further facilitated by the sense‐making work of healthcare professionals, particularly surgeons. A potential implication is that healthcare professionals extend their sense‐making role by relaying the physical and mental health benefits reported by past participants, which may enhance self‐efficacy among patients who feel overwhelmed by their diagnosis, have concerns about the dietary changes, or doubt their ability to adhere to the intervention.

Collective action within the family was interpreted as the main driver of adherence. Supporting qualitative studies exploring experiences with partial and total meal replacement programmes, we found that family support often facilitated adherence through encouraging words, the removal of temptations, or joint participation in self‐directed weight loss [14, 16, 41]. However, our data also contrasts with this literature. While one study reported that participants could ignore family members who encouraged them to eat food outside the meal replacement programme [42], our study found that some families' distrust of the safety of the TDR led some participants not to adhere. It may be that the intervention disrupted families' symbolic use of food to express care and physically fuel the body during cancer, as reported in interviews with families of patients with advanced cancer [43]. We suggest that involving families at the start and throughout the intervention may help mitigate their concerns and encourage them to actively support the participants in adhering to the programme.

Another way that collective action prevented acceptability and adherence was that participants found it difficult adhering to the TDR during social eating occasions, which is a common theme in weight management programmes [14, 19, 44]. Given our corroborating evidence of the importance of the behavioural support for encouragement and problem‐solving [16], the intervention could leverage this resource to help participants substitute social eating occasions that do not involve food. This would help maintain the social cohesion that is important to patients while living with cancer [45].

In line with qualitative studies of physical activity prehabilitation for cancer, there were some reports of side effects, like tiredness, though most participants reported that these were manageable and tended to decrease as they adjusted to the programme [19]. However, unlike these studies where common barriers to adherence included low physical capacity, lack of facilities, other health conditions, and time constraints, such barriers were not reported by the participants here [44]. However, they did report some of these barriers as concerns when we explored whether future interventions could combine physical activity and the TDR. These results suggest that healthcare professionals may want to consider discussing with patients the various prehabilitation options available as a TDR‐only approach may be more suitable for those experiencing these exercise‐related barriers.

Participants were motivated to maintain their weight loss post‐surgery. Some were interested in restarting the intervention after recovery from surgery but also expressed concerns about reverting to old dietary habits. This concern may have been especially salient in this study, where the intervention duration was much shorter than the approximate 2–3‐month period typically used in other disease settings [46]. In addition, participants did not have the opportunity to receive support to practice skills to undertake a phased return to normal food and maintain their lost weight. We suggest that future studies should provide participants with advice on what to expect after stopping the TDR alongside guidance on how to self‐manage their weight after surgery.

## 5 Strengths and Limitations

Strengths included involving a patient representative (P.W.) to review the interview topic guide and interpretation of the findings. This enhanced the credibility of our study by ensuring we asked about experiences that were important to patients and that our interpretations reflected patients' lived experiences. The use of NPT allowed us to go beyond description to explore the theoretical mechanisms influencing the perceived acceptability of the intervention. Future research could explore how these findings compare when analysed with other behaviour and implementation theories. Conducting interviews while the intervention was ongoing might have made it easier for participants to accurately recall their experiences compared to if they had been interviewed after surgery.

Qualitative evaluations in other trials of intervention weight loss as prehabilitation are limited, but our trial's recruitment and adherence rates [17] are consistent to other similar trials conducted in the UK [47], Canada [48], and Australia [49]. In addition, we recruited participants from eight hospitals across England and had consistent findings across age groups and gender. This suggests that populations with different clinical and demographic characteristics and within different healthcare systems may respond relatively consistently to this intervention. Implementation studies are needed to further assess transferability of the findings in real‐world settings as access to prehabilitation may vary.

A limitation affecting the transferability of our findings is that only two of the 31 participants were from the global majority. This reflects a broader limitation of qualitative research within trials, as sampling is restricted to those who choose to enrol in the trial. National programme evaluations of longer TDR programmes in people with type 2 diabetes show that people from Asian, Black, Mixed, or Other ethnic backgrounds have similar uptake but lose 28% less weight than people from White backgrounds [50]. It is, therefore, important that the definitive trial considers strategies to increase sample diversity, paired with testing tailored strategies to improve adherence, and interviews to explore the acceptability of the intervention across ethnic groups.

The number of intervention participants who were not interviewed was relatively low (*n* = 7). The main reason for a lack of interview was non response to our invitation for interview. A qualitative study on a TDR delivered within primary care reported that participants who are less successful with weight loss may be reluctant to share their experiences [16]. In our study, participants who declined to be interviewed lost on average 4.6 kg before surgery, which is 1.5 kg less than the intervention average, corroborating this finding but also suggesting that this group still achieved significant weight loss. While we included a written reflection from a participant who withdrew from the intervention and reports from participants who said they adhered less closely, our findings may not capture potential additional factors that prevented these individuals from losing as much weight.

Acceptability should also be considered in the context of participants who had already demonstrated a baseline level of acceptance by agreeing to join the trial. As typical of qualitative interviews, participants may have presented themselves in a way that was perceived to be more favourable to the researcher.

## 6 Conclusion

In conclusion, an intensive weight‐loss programme with dietetic support as prehabilitation before colorectal cancer surgery was interpreted to be mostly acceptable to people with a BMI of at least 28 kg/m^2^ who had already chosen to join a TDR trial. Participants understood the potential surgical benefits, problem‐solved challenges with the dietitians, and experienced rapid, tangible weight loss that created a positive feedback loop that motivated continued adherence. However, some families' safety concerns, social eating occasions, and side effects prevented full acceptability and total adherence. Addressing these barriers may support more patients to adhere.

## Author Contributions

**Amelia Talbot:** writing – original draft, methodology, writing – review and editing, formal analysis, project administration, data curation. **Claire Foster:** conceptualization, funding acquisition, methodology, writing – review and editing, formal analysis. **Susan A. Jebb:** conceptualization, funding acquisition, methodology, writing – review and editing. **Pete Wheatstone:** funding acquisition, writing – review and editing, methodology. **Simon Buczacki:** writing – review and editing. **Dimitrios A. Koutoukidis:** conceptualization, investigation, funding acquisition, methodology, writing – review and editing, formal analysis, project administration, data curation.

## Ethics Statement

Ethical approval for the CARE trial and this embedded qualitative study was granted by the South Central—Oxford B Research Ethics Committee (Ref: 22/SC/0465).

## Consent

Participants provided fully‐informed written consent.

## Conflicts of Interest

D.A.K. and S.A.J. report being investigators in a publicly‐funded (NIHR) trial where Nestle Health Science and Oviva donated the weight loss intervention to the University of Oxford outside the submitted work. S.A.J. is investigator in investigator‐initiated NIHR‐funded studies, in which Reed Wellbeing and Second Nature have been contracted to provide weight loss interventions outside the submitted work. S.A.J. is co‐investigator outside the submitted work in three investigator‐initiated weight loss trials, the LightCOM programme, funded through a competitive grant from the Novo Nordisk Foundation, an independent philanthropic foundation. In these trials, Liva Healthcare has been contracted to provide the weight loss interventions. Second Nature were the industry partner in an MRC‐funded industrial CASE (iCASE) studentship supervised by D.A.K. and S.A.J. outside the submitted work. The authors have not received personal payments from any company or foundation. No other authors declare conflicts of interest.

## Lived Experience or Public Contribution

People with lived experience were involved in study design, conduct, and interpretation of the CARE. PW has lived experience of colorectal surgery and reviewed the interview questions, attended study meetings, and contributed to our interpretations.

## Supporting information


Supporting File 1

Supporting File 2

## Data Availability

The data that support the findings of this study are available on request from the corresponding author [D.A.K.]. The data are not publicly available as they contain information that compromises the privacy of research participants.
