# Supplementary material for: The Acceptability and Experiences of Participating in a Preoperative Intentional Weight‐Loss Programme From Diagnosis to Surgery for Colorectal Cancer
Source: Health Expect. 2026 Oct 7;29(5):e70896. doi: 10.1111/hex.70896 (PMC13643951; doi:10.1111/hex.70896)
Supplement: Supplementary file 1 [file HEX-29-e70896-s001.docx]

**GUIDE FOR QUALITATIVE INTERVIEW WITH INTERVENTION PARTICIPANTS**

**Topic	Question	Prompt**

Introduction	Thank you for taking the time for the interview. The aim of
the interview is to tell me your thoughts about the diet. We
welcome all comments, positive and negative and indeed
sometimes the negatives comments are the most helpful,
so please feel free to express your views. Can you start by
walking me through how you found the diet when you first
put on it up until now?

Overall view	How are you finding the diet?

Delivery	How do you find your contact with the dietitian?	- Over the phone

contact

- Length of calls

- Knowledge

- Advice and support

Engagement: barriers and facilitators

What has made it difficult to attend the phone calls?	- Work

- Family

- Other commitments

- Feeling unwell

What has helped you to attend the phone calls?	- Improve recovery

- Support by dietitian

How do you keep track of your diet?	- Log of shakes

- Log of weight

- Log of blood

pressure/glucose

How much effort do you feel you need to put in to follow
the recommended diet?

Adherence: barriers and facilitators

If someone was considering starting the diet, what would you say it’s like to follow the diet?

If someone was considering starting the diet, what would you say they need to be aware of?

How has your family and friends found it while you are on the diet?

- Motivation to improve

recovery

- Support by dietitian

- Weight loss

- Ease of use of shakes

- Being part of a trial

- Temptations

- Flavour

- Social occasions

- Side effects

- Approval/disapproval

**Topic	Question	Prompt**

- Positive/negative comments/actions

How have you been feeling during the diet?	- Unhappy, happy

- Different compared

with before starting

How confident are you that you can continue to follow the

diet?

How confident are you that the diet will benefit you?

Have you had any constipation or diarrhoea? If yes, has this [constipation/diarrhoea] worried you?

Have you changed your physical activity since diagnosis?	- Type

- Frequency

- Intensity

- Length

We want to see how we can improve this diet in the future and we would like to ask you your honest opinion on the following.

Losing more weight might improve recovery after surgery to a greater extent. However, that means delaying the surgery for a couple of weeks to lose this weight. If your surgery were to delayed by up to a couple of weeks to lose another 4 pounds/2kilos, would you have been as willing to join the trial?

Symptoms of both TDR and cancer

Physical activity

Future research questions

| We would like to test how this diet works together with physical activity. Now that you know how it feels to be on this diet, how would you feel if in addition to the diet, we had asked you to increase your physical activity?  How would you feel about re-starting the diet a couple of months after you recover from surgery to help you lose more weight and improve your long-term health?  Close down Any further comments? Thank you for taking part in the interview. | If positive  - type of activity e.g.,  walking, resistance  If negative  - why not, hunger, asking “too much”, too tired |
| --- | --- |
