# Supplementary material for: The Acceptability and Experiences of Participating in a Preoperative Intentional Weight‐Loss Programme From Diagnosis to Surgery for Colorectal Cancer
Source: Health Expect. 2026 Oct 7;29(5):e70896. doi: 10.1111/hex.70896 (PMC13643951; doi:10.1111/hex.70896)
Supplement: Supplementary file 2 [file HEX-29-e70896-s002.docx]

*Illustrative Quotes for Construct One: Coherence*

| **Construct One: Coherence**  The sense-making work to understand the purpose and potential benefits of the intervention. | |
| --- | --- |
| **Component** | **Supporting Quote(s)** |
| **Individual Specification:**  What was the participants’ understanding of what they needed to do as part of the intervention? | “*This is week four. I finish on Saturday because I've got my operation on Tuesday*.” (female, 56 years, BMI 33.4, -6.7kgs)  “*I take them at a certain hour… Normally I take two soups, a porridge, and a shake.”* (female, 54 years, BMI 34.8, -7.9kgs) |
| **Internalisation:** What was the participants' understanding of the value and importance of the intervention? | “*It's knowing that I've got to give myself the best chance when I have the operation on the cancer. And if this* [TDR] *is going to help, then so be it*.” (male, 69 years, BMI 37.6, -6.6kgs)  *“No doubt he* [surgeon] *hasn't got so much blubber to go through to get where he needs to be… I'm sure it's better for my heart not to be carrying so much around while I'm recovering. I've got to get mobile quickly after the operation, maybe the following day*.” (female, 64 years, BMI 40.3, -5.6kgs) |
| **Differentiation:**  How did participants differentiate the intervention from their usual dietary habits and/ or other diets? | "*In the first week, I lost 4 kilos. I weighed in at 92kg last week, and then this week, I'm down to 89 kg. It's slowed down… but to lose 2 kilos a week is amazing. I've had diets before, and in the first week, you lose half a stone. The second week, you might lose a pound, and you get disheartened, and then you might put a little bit on, and you think, 'hang on a minute. I hadn't done anything differently. I've stuck to the same diet, but I put weight on now,' and then I've gone back to eating normal food. But this one* [TDR] *I've really stuck to, and I am losing weight*." (female, 62 years, BMI 33.9, -12.2kgs) |

*Illustrative Quotes for* *Construct Two: Cognitive Participation*

| **Construct Two: Cognitive Participation**  The relational work to build and sustain motivation and adherence to the intervention. | |
| --- | --- |
| **Component** | **Supporting Quote(s)** |
| **Initiation:**  What were the actions taken by healthcare providers to promote participants' initial engagement with the intervention? | “*I was really looking forward to the diet beforehand, hoping I'd lose some weight because the surgeon said I'm definitely overweight*.” (male, 74 years, BMI 33.9, -2.9kgs)  “*I felt optimistic that I would lose some weight* [while following the intervention] *because my surgeon wanted me to lose weight to improve my chances with the operation and improve my recovery*.” (female, 64 years, BMI 40.3, -5.6kgs) |
| **Activation:**  What were the ongoing actions made by participants with their healthcare providers to maintain their motivation and adherence to the intervention? | “*You're telling somebody* [dietitian] *how you're getting on, not just talking with the wife and saying ‘I'm not finding this very easy’ and her saying, ‘Well, it doesn't look that good to me’ At least you're talking to somebody different and getting the feedback.”* (male, 74 years, BMI 33.9, -2.9kgs)  “*We had a long chat over the phone, and I did say, ‘I’m thinking that I might give up,’ and she was quite supportive and said, ‘Yeah, I can understand why, but you know, these are the advantages of continuing*.” (male, 66 years, BMI 30.2, -2.2kgs)  “*The other advice* [from the dietitian] *has been to answer questions. Such as the order in which I take things, the timings, and the composition. You know, if I feel like it, can I have four strawberry shakes in one day or something? They* [dietitian] *said it nutritionally it doesn't matter which I take*.” (male, 65 years, BMI 42.6, -9.6kgs) |

*Illustrative Quotes for Construct Three: Collection Action*

| **Construct Three: Collection Action**  The operational work to enact the intervention into everyday practice. | |
| --- | --- |
| **Component** | **Supporting Quote(s)** |
| **Interactional Workability:**  How did participants' interactions with the intervention enable or hinder continued adherence? | “*It is very little effort once you get the water into the right level, shake it up, it's easy*.” (female, 62 years, BMI 48.3, -5.3kgs)  “*I've had no problems with it* [the intervention]*. I don't feel hungry. I did think I'd be hungry. But I haven't done at all*.” (female, 77 years, BMI 40.8, no weight loss data due to withdrawal) |
| **Contextual Integration:**  How did participants find that the intervention integrated into their social and environmental contexts? | “*Socially, it's the death, really, isn't it? What we* [friends] *would normally do is a couple of gin and tonics and a nice meal. I'm not able to do that at the moment.”* (female, 64 years, BMI 40.3, -5.6kgs)  "*The first couple of weeks were fairly difficult because we were going out a lot, and we were with people who were eating. We've* [family and participant] *avoided that for the last few weeks, and that has made it a lot easier. You've not got that temptation. You haven't got that constant reminder in front of you*." (male, 61 years, BMI 32.4, -7.8kgs) |
| **Relational Integration:** How was the knowledge to support adherence to the intervention mediated within participants' social networks? | “*My wife's kind of joined me on a diet as she bought some new off-the-shelf protein shake and doing the same sort of thing. That has been helpful*.” (male, 63 years, BMI 38.9, -9.4kgs)  "*My husband keeps saying I think you've been on it too long. And I said, 'Oh well, you know, I don't care what you think'*." (female, 59 years, BMI 35.8, -5.5kgs) |

*Illustrative Quotes for Construct Four: Reflexive Monitoring*

| **Construct: Reflexive Monitoring**  The appraisal work to evaluate whether the intervention had been beneficial and effective. | |
| --- | --- |
| **Component** | **Supporting Quote(s)** |
| **Individual Appraisal:**  How did participants evaluate the effectiveness of the intervention? | “*When the numbers* [weight] *started coming down, I really did have a lot more energy, even just like, it sounds really silly, just standing up and just going somewhere quite quickly as if I was a lot younger*.” (female, 59 years, BMI 35.8, -5.5kgs)  “*I write it down* [weight loss at completion of a day] *as I've got a time on a calendar on the wall. When I started the first day, I put the first cross on to say I've done a day. I can see the build-up of time, and I also put my weight once a week on there, so I can see that it’s gone down South*.” (female, 72 years, BMI 34.0, -5.1kgs)  “*My worry* [about surgery] *was having a stoma protruding because your tummy is big, and then you've got a stoma, and people are going to see it. So I'd have to buy very baggy clothes… So when this trial came about, the first thing I remember thinking was, ‘I'm gonna lose weight around my tummy’ because I know that's where my weight goes fast. Even if I have a stoma…, then I don't have to change the size of my clothes. When I started the fourth week, I knew that was going to happen.* (female, 54 years, BMI 34.8, -7.9kgs) |
| **Systemisation:** What data analytic processes did participants engage in to assess the effects of the intervention? | “*It didn't really seem to bother my diabetes checks. I've always been under 10 mmol/L* [since starting the TDR], *and before I started the diet, I should imagine I was in the tens to 20 mmol/L. I find it quite amazing because I thought it would be a bit drastic to alter my Humulin* [insulin medication] *to half on the day I started* [the intervention].” (female, 59 years, BMI 35.8, -5.5kgs)  “*My blood pressure has come down while we've been doing it, so, you know, it's all been positive, really*.” (female, 64 years, BMI 40.3, -5.6kgs) |
| **Reconfiguration:**  What were the participants' recommendations on how the intervention could be modified to improve its acceptability? | “*It gets a bit boring after a while. I'm quite glad that I'm coming to the end of it. I mean, there are three soups, and it gets a bit monotonous. Another couple of flavours of soup would relieve the monotony a bit*.” (female, 61 years, BMI 34.7, -5.0kgs)  “*Yes* [I would take the TDR after recovering from surgery]*, I'd like to lose more weight, but whether or not I could actually* [adhere]*, because it is so restrictive. I probably would want something slightly less restrictive than that diet to be able to actually have a social life as well as lose weight*.” (male, 61 years, BMI 32.4, -7.8kgs)  “*If I'm honest, no* [I would not delay my surgery beyond four weeks]*. Because you never know what can happen with the tumour. You don't know if it's going to grow more*” (female, 54 years, BMI 43.1, -6.1kgs) |
